# The role of growth factors in human sperm parameters: A review of in vitro studies

**DOI:** 10.18502/ijrm.v20i10.12265

**Published:** 2022-11-02

**Authors:** Hanieh Ghasemian Nafchi, Yaser Azizi, Iman Halvaei

**Affiliations:** ^1^Student Research Committee, Iran University of Medical Sciences, Tehran, Iran.; ^2^Physiology Research Center, Iran University of Medical Sciences, Tehran, Iran.; ^3^Department of Physiology, Iran University of Medical Sciences, Tehran, Iran.; ^4^Department of Anatomical Sciences, Faculty of Medical Sciences, Tarbiat Modares University, Tehran, Iran.

**Keywords:** Growth factors, Sperm, ROS, Cryopreservation, In vitro.

## Abstract

In vitro sperm preparation/incubation and cryopreservation are associated with oxidative stress as the main cause of sperm damage, and different strategies are used to improve sperm quality in in vitro conditions to treat male infertility. Growth factors (GFs) are biological molecules that play different roles in various cellular processes such as growth, proliferation, and differentiation. Many studies have shown that GFs and their receptors are expressed in the male reproductive system. In vitro supplementation of GFs to improve sperm parameters has yielded useful results. There are many studies on the effects of GFs on sperm quality improvement and subsequent assisted reproductive technology results. Hence, this study will review the in vitro results of various GFs including brain-derived neurotrophic factor, nerve growth factor, fibroblast growth factor, insulin-like growth factor I, and vascular endothelial growth factor to improve sperm quality.

## 1. Introduction

Infertility affects about 15-20% of couples worldwide, of which 50% are due to male factors (1). Reactive oxygen species (ROS) are major causes of sperm DNA damage and subsequent male infertility (2). Endogenous sources of ROS in the seminal plasma include leukocytes, immature spermatozoa, and varicocele, and exogenous sources also include radiation, toxins, tobacco, and alcohol (3). Assisted reproductive technology (ART) has been introduced as a set of techniques and methods for the treatment of infertility. The first step in ART is sperm preparation, in which the spermatozoa are separated from non-sperm cells and seminal fluid. One of the inevitable steps in routine sperm processing is centrifugation. It was shown that multiple centrifugations could generate a high level of ROS and cause sperm DNA damage (4). Temperature fluctuation during ART is also one of the factors producing ROS. Visible light (400-700 nm) is another inducer of ROS production that impairs gamete and embryo development (5). In vitro manipulation exposes the sperm to visible light from microscopes and laboratory (6). In addition, sperm cryopreservation has been a routine procedure in ART clinics. It has been reported that the cryopreservation process causes harmful changes to the sperm, and temperature changes during cryopreservation might also lead to the production of ROS and subsequent oxidative stress (7, 8).

When the quality of spermatozoa is very low in severe male factor infertility, the spermatozoa are more vulnerable to different damages. The quality of spermatozoa in vitro could be improved using different strategies. Different drugs improve sperm parameters with possible side effects (9). Using growth factors (GFs) in vitro has been introduced to improve sperm parameters in infertile patients because they are present in the testes and semen. Sometimes short incubation of spermatozoa with GFs improves sperm parameters (10). GFs are important for the regulation of various cellular processes, including growth stimulation, proliferation, and cell differentiation. These include nerve growth factor (NGF), brain-derived neurotrophic factor (BDNF), fibroblast growth factor (FGF) 2, vascular endothelial growth factor (VEGF), insulin-like growth factor-I (IGF-I), and platelet-rich plasma (PRP). It was shown that GFs have receptors in the reproductive tissues, revealing their roles in the reproductive processes (11, 12).

The beneficial effects of adding GFs on sperm parameters have been shown previously (13). Many investigators have focused on using GFs in the ART to improve sperm quality and subsequent reproductive outcomes (13, 14). GFs may have different effects via different mechanisms on sperm parameters and functions that make them good candidates for different applications in the future. GFs could be added in the sperm preparation/cryopreservation medium to ameliorate detrimental effects of incubation/cryopreservation but there is no standard protocol in this regard. There are few studies that reviewed the effects of in vitro supplementation of different GFs on human spermatozoa. We aimed to review different in vitro supplementation of GFs for human spermatozoa, focusing on findings and probable mechanisms.

## 2. Materials and Methods

We conducted a comprehensive search of electronic databases including PubMed, Web of Science, Embase, Scopus, and Google scholar from 1980-2021. The following terms were searched in titles and abstracts: (“sperm” OR “spermatozoon” OR “spermatozoa” OR “sperm cell” OR “sperm cells” AND (“growth factor” OR “growth factors”) AND (“cryopreservation” OR “cryofixation” OR “cryonic suspension” OR “freezing” OR “freeze-thaw process” OR “post-thaw” OR “frozen-thawed” OR “rapid freezing” OR “slow freezing” OR “vitrification” OR “chilled storage”)/(“sperm incubation” OR “incubation”. The references of included articles were also studied.

## 3. Results

### Sperm incubation with GFs

#### Neurotrophins

Neurotrophins are a family of proteins expressed in the central nervous system and are essential for survival, evolution, differentiation, and neuronal function. The family of neurotrophins includes NGF, neurotrophin-4/5, BDNF, and neurotrophin-3 (15). The expression of neurotrophins in the reproductive tissues suggests that they can be involved in spermatogenesis (16).

#### BDNF

It has been shown that BDNF may have antioxidant properties (17), and adding a BDNF supplementation to the sperm preparation medium has been able to improve human sperm function (18). Najafi et al. showed that incubation of normozoospermic samples with 0.133 nM BDNF for 60 min significantly improved sperm motility and decreased the percentage of immotile sperm. They also showed the mean fluorescence intensity of 2'-7' dichlorofluorescin diacetate as an indicator of intracellular ROS, which significantly decreased in sperm incubated with BDNF. In addition, BDNF significantly reduced apoptosis and increased the percentage of viable sperm cells (18). Safari and colleagues showed that incubation of normozoospermic samples (male age: 22-40 yr) with 0.133 nM BDNF for 60 min had beneficial effects on sperm parameters. They showed that BDNF improved sperm motility and viability. Also, it was found that in the groups treated with BDNF, the levels of lipid peroxidation and nitric oxide (NO) significantly reduced. In addition, the mitochondrial activity improved, and secretion of insulin and leptin increased. BDNF has also been shown to reduce NO levels leading to a decrease in the caspase range and preventing apoptosis in spermatozoa (19).

#### NGF

It was shown that incubation of normozoospermic samples with 10 µM NGF for 30 min significantly increased progressive motile sperm compared to the control group. It was also reported that NGF at 1 µM and 10 µM increased sperm movement distance and the rate of progressive motility compared to 0.1 µM in a dose-dependent manner. Also, an increase in sperm movement distance along with an increase in incubation time has been observed (20). The beneficial effects of adding 0.5 ng/mL and 5 ng/mL NGFβ in the culture medium during sperm processing were recently shown. They showed that incubation of 10^6^ spermatozoa after density gradient centrifugation at 37°C significantly increased progressive motility and viability compared to the control group (10).

#### FGFs

Garbarino Azúa et al. reported that incubation of normozoospermic samples in the age range of 28-50 yr with 10 ng/mL recombination FGF2 for 30 min significantly improved sperm motility compared to 0 or 100 ng/mL groups. They also showed the presence of FGF2 in human spermatozoa (14). In addition, it was reported that sperm incubation with 100 ng/mL FGF2 for 2 hr significantly improved sperm motility compared to the controls (55 ± 4 vs. 42 ± 4%, respectively), and incubation with 10 ng/mL only increased grade a motility (21).

#### VEGF

Iyibozkurt and co-workers reported that VEGF had the best effect at a dose of 15 ng/mL on sperm motility and linear velocity. Still, no significant differences were observed in sperm viability after 24 hr of incubation with any concentration of VEGF in the semen samples of fertile men. They showed that the addition of VEGF improved sperm motility and the presence of VEGF receptors on sperm indicated direct effects of VEGF on spermatozoa (22).

#### IGF-I

Asimakopoulos and colleagues recently evaluated the possible effects of the incubation of human spermatozoa using IGF-I. They found that incubation after 1 hr at 37°C with 2 concentrations of 100 ng/ml and 1000 ng/ml IGF-I improved sperm motility and viability. In comparison with NGF, they also showed that IGF-I was more effective (10).

#### PRP

Bader et al. reported that in both non-stressed and stressed (caused by hydrogen peroxide (H ${}_{2}$ O ${}_{2}$ )) spermatozoa of healthy men with the mean age of 35 ± 5 yr, incubation with 2% PRP for 24 hr significantly improved sperm motility and viability, decreased DNA fragmentation, vacuolization, and ROS-positive cells. But the sperm morphology was the same between different groups (23).

### The supplementation of GFs in sperm cryopreservation

#### NGF

Saeednia et al. reported that adding 0.5 ng/mL NGF to the freezing medium in semen samples from normozoospermic men significantly improved sperm motility, viability, intracellular NO concentration, and decreased apoptosis in spermatozoa (24). The same group showed that adding 0.5 ng/mL NGF to the freezing medium in asthenozoospermic samples significantly improved sperm motility, viability, and reduced DNA fragmentation. Still, NGF at all concentrations (0.5, 1, 5 ng/mL) could not change the NO content (25).

#### BDNF

It was shown that the use of BDNF supplementation (0.133 nM) in semen samples from fertile men in the freezing or thawing medium significantly improved sperm motility and viability, decreased intracellular H ${}_{2}$ O ${}_{2}$ , superoxide anion concentration (O2 ${}^{-}$ ) and caspase-3 activity, and increased AKT phosphorylation. Therefore, supplementation of the sperm freezing and thawing medium with BDNF improved sperm survival, motility, and fertilization potential after cryopreservation and protected the sperm against oxidative stress and apoptosis caused by cryopreservation procedure (13).

#### PRP

Yan et al., very recently, reported that adding autologous 5% PRP to normozoospermic samples improved sperm viability, progressive motility, and membrane integrity after cryopreservation. In addition, PRP affected the mitochondrial membrane potential (MMP) recovery and decreased ROS production and DNA fragmentation, but this difference was not significant (26). A summary of the studies is shown in table I.

**Table 1:** In vitro effects of GFs addition on human sperm


			
**Author, yr (Ref)**	**Type of growth factor**	**Dosage**	**Sample**	**Manipulation**	**Findings**
**Najafi ** ***et al.*** **, 2017** **(18)**	BDNF	0.133 nM	Normospermia	Incubation	↑ Motility ↓ ROS ↓ Apoptotic-like change
**Safari ** ***et al.*** **, 2018** **(19)**	BDNF	0.133 nM	Normospermia	Incubation	↑ Motility ↑ Viability ↑ Mitochondrial activity ↓ LPO ↓ NO ↑ Insulin and leptin secretion
**Lin ** ***et al.*** **, 2015 (20)**	NGF	1 µM 10 µM	Normospermia	Incubation	↑ Motility
**Asimakopoulos** ***et al.*** **, 2021 (10)**	NGFβ	0.5 ng/mL 5 ng/mL	Normal and abnormal (severe oligo-, astheno-, or teratozoospermia excluded)	Incubation	↑ Motility ↑ Viability
**Garbarino Azúa ** ***et al.*** **, 2017 (14)**	rFGF2	10 ng/mL	Normospermia	Incubation	↑ Motility
**Saucedo ** ***et al.*** **,** **2015 (21)**	FGF2	10 ng/mL 100 ng/mL	Donor (NA)	Incubation	↑ Motility ↑ Motility (grade a)
**Iyibozkurt ** ***et al.*** **,** **2009 (22)**	VEGF	10 ng/mL 15 ng/mL	Normospermia	Incubation	↑ Motility
**Asimakopoulos** ***et al.*** **, 2021 (10)**	IGF-I	100 ng/mL 1000 ng/mL	Normal and abnormal (severe oligo-, astheno-, or teratozoospermia excluded)	Incubation	↑ Motility ↑ Viability
**Bader ** ***et al.*** **,** **2020 (23)**	PRP	2%	Normospermia	Incubation (Non-stressed and stressed spermatozoa)	↑ Motility ↑ Viability ↓ DNA fragmentation ↓ Vaculization ↓ ROS
**Saeednia ** ***et al.*** **,** **2015 (24)**	NGF	0.5 ng/mL	Normospermia	Cryopreservation	↑ Motility ↑ Viability ↑ Intracellular NO ↓ Apoptosis
**Saeednia ** ***et al.*** **,** **2016 (25)**	NGF	0.5 ng/mL	Asthenozoospermia	Cryopreservation	↑ Motility ↑ Viability ↑ DNA integrity
**Najafi ** ***et al.*** **,** **2016 (13)**	BDNF	0.133 nM	Normospermia	Cryopreservation	↑ Motility ↑ Viability ↓ H ${}_{2}$ O ${}_{2}$ and O ${}_{2-}$ ↓ Caspase activity ↑ AKT phosphorylation
**Yan ** ***et al.*** **, 2021** **(26)**	PRP	5%	Normospermia	Cryopreservation	↑ Motility ↑ Viability ↑ Membrane integrity
BDNF: Brain-derived neurotrophic factor, NO: Nitric oxide, NGF: Nerve growth factor, NGFβ: Nerve growth factor beta, rFGF2: Recombinant fibroblast growth factor 2, VEGF: Vascular endothelial growth factor, IGF-I: Insulin-like growth factor-I, PRP: Platelet-rich plasma, LPO: Lipid peroxidation, ROS: Reactive oxygen species, NA: Not available, ↑ : Increase, ↓ : Decrease					

## 4. Discussion

In vitro supplementation of GFs in andrology could be a good choice for improving treatment outcomes with different mechanisms, especially in abnormal cases, but there remains many unanswered questions about how to use GFs to achieve optimal results. The optimum dose should be defined for each GF and excessive application is not recommended. It should be noted that the protective effects of GFs on the sperm are via antioxidant properties and adding a high dose of GFs would be detrimental because of reductive stress (27). Personalized antioxidant used in infertility treatment might be available in the future. Different aspects of the patient and specimen including male age, lifestyle, specimen type (normal or abnormal) should be taken into account when choosing the type and dose of GF (28). Most studies have evaluated sperm motility and viability following sperm incubation with GFs, although there is a lack of data on the probable effects of GFs incubation on DNA integrity and chromatin packaging that may be due to the short incubation time. Other GFs like epidermal growth factor (29), transforming growth factor-β (TGF-β) 1 were used to improve animal sperm parameters, which make them good candidates for in vitro supplementation for human spermatozoa (30). One of the discrepancies in the literature is different incubation times. It is also necessary to find the optimum incubation time to get the best results.

Regarding possible adverse effects of adding GFs on physical and chemical characteristics of sperm culture media, it should be noted that adding different GFs may not affect pH levels because pH is related to the concentration of hydrogen ions in the solution and GFs have no effects on hydrogen concentration. One of the most important factors that determines the osmolarity of solution is solution molarity and the number of ions. There are no reports on the detrimental effects of adding GFs on the osmolarity of the sperm culture medium. Indeed, GFs (like VEGF) are present in semen per sec, (31) and compounds found in the reproductive system (like oviduct) can be isolated and added into the sperm culture media to mimic the biochemical composition of the reproductive system (32). Besides, Yan et al. reported that PRP, as a source of GFs, might have protective effects against osmotic shock and thus might protect cell membranes during various stages of the cryopreservation process (26).

### BDNF

BDNF, as a member of the neurotrophin family, is expressed in the nervous system and is involved in the processes of differentiation, survival, maturation, and regeneration of neuronal cells. It has been reported that BDNF played an important role in the male reproductive system (15, 16), and its receptor was found in spermatozoa (33). It was shown that BDNF mRNA and protein were expressed in different parts of human spermatozoa, including the head, neck, and tail. Abnormalities in the *BDNF* gene expression might be associated with the pathogenesis of male infertility disorders and the expression of BDNF in the semen sample of oligoasthenozoospermic men is lower than infertile men (15). It was also found that the tyrosine kinase Trk receptor (BDNF receptor) was expressed in human spermatozoa (18). The protective effect of BDNF against oxidative damage might be discovered by modifying antioxidant enzymes. Valvassori et al. reported that BDNF might decrease superoxide dismutase (SOD) activation and increase catalase enzyme activity (34). It has also been proposed that the protective effect of BDNF was due to increased phosphorylation of the cyclic adenosine monophosphate response element-binding protein and subsequent suppression of cytochrome C released to the cytosol (34). In addition, it has been shown that the induction of Sestrin 2 gene expression could explain the antioxidant effect of BDNF. Another reason for the antioxidant activity of BDNF is probably due to its ability to modulate antioxidant enzymes or scavenge free radicals (35). However, other researchers have indicated that BDNF reduced apoptosis in several pathways, such as phosphatidylinositol 3 kinase (PI3K)/AKT (protein kinase B) pathway activation (36), increased the expression of Bcl-2 anti-apoptotic protein, and reduced activation of caspases-2 and -3 (37). Besides, some studies have concluded that AKT maintained sperm parameters, including motility, viability, and MMP, by inhibiting the activation of caspases-3 and -7 (38). It has also been reported that apoptosis occurred by inhibiting PI3K activity leading to rapid motility loss and oxidative DNA damage (39). BDNF is involved in PI3K pathway activation and may play an important role in improving motility and DNA integrity through this pathway (36).

The role of insulin and leptin in increasing PI3K activity in sperm has been shown previously (40). BDNF is likely to play a role in insulin and leptin secretion, thereby improving sperm motility and survival rate (19). It has been also suggested that BDNF regulated sperm metabolism by affecting mitochondrial potential (33).

The protective effect of BDNF against oxidative stress and apoptosis, as a consequence of the cryopreservation procedure, may be activated through transient activation of the PI3K/AKT pathway (13). It seems that the protective effect of BDNF against oxidative damage is probably through the modulation of antioxidant enzymes or free radicals scavenging (35). BDNF and vitamin D supplementation have also been shown to have protective effects in cerebellar granule cells through various mechanisms such as MMP, Bax translocation, and reduction of ROS generation (17).

### NGF

Pro-NGF contains NGFα, NGFβ, and NGFγ in a ratio of 2:1:2 (10). As one of the most important neurotrophic factors, NGF plays a very important role in the growth, development, structural and functional maintenance, and repair of the nervous system. NGF performs its role through 2 receptors: tropomyosin kinase A (TrkA) and a receptor with low affinity called low-affinity NGF receptor/p75 neurotrophin receptor (20, 41, 42). Previous studies have shown the presence of NGF and its receptors in the testis, epididymis, and sperm, indicating its possible role in the reproductive system and regulation of sperm function (12, 43).

Seidl et al. in their mouse study confirmed the expression of NGF in the Sertoli, Leydig, and myoid cells in the testis and different distribution of its receptor; TrkA was present in non-germ cells, and p75 was expressed in Sertoli and myoid cells (44). Subsequent studies also verified NGF expression and its receptors in the testis and different germ cells in rats, monkeys, hamsters, and bovine (12, 45-47). Robinson et al. revealed the expression of neurotrophins and their receptors in the human fetal testis (48). The next study showed the expression of NGF in the adult human testis as well (49). Li et al. found that NGF in semen and TrkA mRNA in spermatozoa were lower in oligoasthenozoospermic samples compared to samples from fertile and asthenozoospermic men (50). It seems that binding of NGF to its receptor (TrkA) leads to ligand-induced dimerization, which increases autophosphorylation of the tyrosine kinase segment, followed by activation of the RAS/MAPK and PI3K/AKT pathway (51). AKT can maintain sperm motility, viability, and MMP by inhibiting the activation of caspases-3 and -7 (38).

Several mechanisms have been studied regarding the protective effects of NGF. It was reported that exogenous NGF could improve the cells' survival through Trk receptor protein (12).

The antiapoptotic effect of NGF has been reported in various cells. NGF may affect sperm DNA integrity by activating the PI3K pathway and inhibiting downstream apoptotic signaling such as caspase-3 rotenone-induced activation in dopaminergic cells (52) or by upregulating the phosphorylation of the Trk receptor. NGF can protect hippocampal progenitor cells from apoptosis against staurosporine toxicity by affecting caspase-3 or upstream activation (53). Therefore, it seems that the effect of NGF on sperm DNA integrity is activated through these pathways.

### FGFs

FGFs and their receptors are involved in many cellular processes, including the growth and maintenance of normal tissues. FGF2 is an important member of the FGFs family, and 5 human FGF2 isoforms (18, 22, 22.5, 24, and 34 k Da) have also been identified (54).

The presence of FGFs and their receptors in the male reproductive tissues and their roles in regulating reproductive performance have been confirmed (11). Recently it has been shown that FGFR1 mutations were related to lower sperm concentration (55).

In somatic cells, the activation of FGF/FGFR signaling pathways plays a role in activating Ras/ERK and PI3K/AKT pathways (56), and it should be noted that some components of these pathways, such as ERK1/2, PI3K, and AKT, play an important role in maintaining the function of mammalian sperm. The presence of ERKs in the equatorial segment of human spermatozoa is probably related to regulating proteins that mediate binding between sperm and egg plasma membranes (57). Also, the role of the ERK pathway in maintaining sperm motility has been reported (58). FGF2 increases ERK and AKT phosphorylation, and it seems that the effect of FGF2 on sperm motility is mediated by FGFR activation as pre-incubation with BGJ398 (a potent and selective pan-FGFR antagonist) eliminate this effect (21).

### VEGF

VEGF is an important polypeptide involved in the angiogenesis process. VEGF and its receptors in the male reproductive system have been confirmed, and the VEGF protein is present in the spermatids, seminal plasma, Sertoli, and Leydig cells (59). It was shown that VEGF could prevent oxidative damage by activating the nuclear factor erythroid 2-related factor 2 pathway in vitro (60). VEGF was also associated with mitochondrial biogenesis via serine-threonine kinase protein kinase B (AKT) (61). Also, in the nervous system, maintenance of MMP and regulation of Bcl-xL and cytochrome C release are associated with AKT pathway activation (62). Therefore, VEGF affects sperm motility by maintaining MMP through the AKT pathway (22).

### IGF-I

IGF-I is a 70 aa hormone whose structure is 50% similar to proinsulin. Liver is the main organ that produces IGF-I in response to growth hormone, and it can be secreted by every tissue for paracrine and autocrine functions (63, 64).

Three receptors have been found in IGF-I, including the IGFR-I receptor, insulin receptor, and IGFR-I-insulin receptor (hybrid) (63). The presence of IGF-I in testis, germ cells, and seminal plasma has been shown previously in human samples (65-67). It was demonstrated that the IGF-I receptor was not present in the plasma membrane of sperm from patients with a history of fertilization failure (68). IGF-I in seminal plasma was correlated with semen quality (66).

IGF-I may improve sperm motility by different mechanisms. Also, it could be considered an antioxidant (69). IGFR-I receptor is the main IGF-I receptor that activates the AKT pathway with several functions, such as affecting FOXO, p53, cyclic monophosphate response element-binding protein, and nuclear factor kappa-light-chain-enhancer of activated B cells (NF-KB) leading to survival gene expression (63).

### PRP

PRP is a biological product that has been used in various medical fields such as dermatology, orthopedics, and dentistry (70). PRP is derived from a patient's blood sample and contains plasma with a platelet concentration higher than normal blood. Its positive effects come from having various factors such as VEGF (22), FGF (21), SOD (71), TGF-β, platelet-derived growth factor, and zinc (72, 73). The levels of TGF-β2 and TGF-β3 in semen are related to fertility status (74).

The beneficial effects of PRP are related to its various components. It has been reported that incubation of goat spermatozoa with IGF-1 significantly reduced sperm DNA fragmentation (75).

The molecular mechanism of IGF-I has been shown to regulate the function and survival of various cells by regulating the mitochondrial cytochrome C/caspase pathway (76). Recently, seminal plasma IGF-I was found to be associated with sperm motility in infertile patients (65), and methylation levels of *IGF-2* in spermatozoa from infertile men may be associated with sperm DNA fragmentation (77). The antioxidant zinc/copper SOD enzyme is an important component of PRP. As one of the components of the ROS scavenger system, by inhibiting the lipid peroxidation of human, spermatozoa reduce DNA fragmentation caused by H ${}_{2}$ O ${}_{2}$ exposure resulting in an increase in sperm motility (23, 71, 78). Therefore, a combination of these factors in PRP can reverse the adverse effects of H ${}_{2}$ O ${}_{2}$ -induced oxidative stress and play an important role in improving sperm parameters and subsequently male infertility (23).

## 5. Conclusion 

Cryopreservation and sperm manipulation are necessary and practical techniques in infertility management, but they may affect sperm parameters, including motility, viability, and DNA integrity. Supplementing the sperm culture/cryopreservation medium with different GFs may improve human sperm parameters. Therefore, they minimize the detrimental effects of manipulation techniques. Besides, abnormal spermatozoa could benefit more than normal samples, and due to the antioxidant potential of GFs, these factors can be used for abnormal samples with high ROS production and during the freeze-thaw process. Using GFs in sperm processing and/or incubation with spermatozoa can be used in ART, especially in intrauterine insemination or conventional in vitro fertilization to improve sperm motility and viability. GFs used in the literature have shown satisfactory results, but more future studies remain necessary. Further studies should be performed to assess the clinical application of different types of GFs on normal/abnormal sperm samples to draw a standard protocol. Investigating molecular mechanisms of different GFs affecting sperm function helps clinicians treat infertile/subfertile men.

##  Conflict of Interest 

The authors declare that there is no conflict of interest.
